# Assessment of left atrial function in patients with type 2 diabetes mellitus with a disease duration of six months

**DOI:** 10.5830/CVJA-2017-048

**Published:** 2018

**Authors:** Gulmez Oyku, Parildar Hulya, Cigerli Ozlem, Demirağ Nilgun

**Affiliations:** Department of Cardiology, Baskent University, Istanbul Medical and Research Centre, Istanbul, Turkey; Department of Family Medicine, Baskent University, Istanbul Medical and Research Centre, Istanbul, Turkey; Department of Family Medicine, Baskent University, Istanbul Medical and Research Centre, Istanbul, Turkey; Department of Endocrinoloy and Metabolism, Baskent University, Istanbul Medical and Research Centre, Istanbul, Turkey

**Keywords:** left atrial volume, left atrial function, diabetes mellitus, transthoracic echocardiography

## Abstract

**Introduction:**

Changes in left atrial (LA) size and function are associated with adverse clinical events. Recently, duration of diabetes mellitus (DM2) has been found to be positively associated with increased LA volume and impaired LA function. This study was performed, using two-dimensional echocardiograpy, to evaluate the changes in LA volume and function in patients with DM2 with a disease duration of six months, and to assess the parameters that affect LA volume and function.

**Methods:**

Fifty-six patients (28 male, age: 52.6 ± 6.5 years) with DM2 and 56 controls (24 male; age: 50.1 ± 7.0 years) were enrolled in the study. Each subject underwent conventional twodimensional echocardiography to assess LA volume (indexed maximal LA volume: Vmax, pre-atrial contraction volume: Volp, minimal LA volume: Vmin) and LA function [passive emptying volume – passive emptying fraction (PEV – PEF), active emptying volume – active emptying fraction (AEV – AEF), total emptying volume – total emptying fraction (TEV – TEF)].

**Results:**

LA diameter, indexed Vmax, Volp, Vmin, AEV and TEV were found to be significantly higher in the DM2 group compared with the controls (p < 0.05). Indexed Vmax, Volp and Vmin were significantly correlated with HbA_1c_ level, body mass index (BMI), high-sensitivity C-reactive protein and uric acid levels, mitral A wave, E/E′ ratio and A′ wave. According to multivariate analysis, age and BMI had a statistically significant effect on LA volume.

**Conclusion:**

Impaired LA function may be present in patients with newly diagnosed DM2. BMI and increasing age caused LA enlargement and LA volumes that were independent of the effects of hypertension and DM2.

The prevalence of type 2 diabetes mellitus (DM2) increases over a person’s lifetime due to aging, the epidemic of obesity and sedentary lifestyles. Moreover, the incidence of cardiovascular disease (CVD), and morbidity and mortality due to CVD increase in patients with DM2.^1^,^2^

Early changes in left ventricular (LV) function in patients with DM2 have been extensively investigated, however, assessment of left atrial (LA) function is of growing interest.^2^-^8^ The left atrium serves as a reservoir during ventricular systole, as a conduit during early diastole, and as an active contractile chamber that augments LV filling in late diastole.

Total emptying volume (TEV) describes LA reservoir function, passive emptying volume (PEV) describes LA conduit function, and active emptying volume (AEV) describes LA booster pump function.^7^,^9^ Two-dimentional (2D) echocardiography is a non-invasive, easy-to-use and accessible method to evaluate LA volume and function.

Several studies have shown that changes in LA size and function were associated with adverse clinical events such as atrial fibrillation, stroke, diastolic dysfunction and LV failure.^10^-^13^ Moreover, studies that evaluated LA volume and function in patients with DM2 showed that LA volume and function were independent predictors of cardiovascular events.^4^-^8^ Recently, the duration of DM2 disease has been found to be strongly and positively associated with larger LA volume and impaired LA function measured by echocardiography.^14^

The aims of our study were to evaluate the change in LA volume and function, and assess the parameters that affect LA volume and function in patients with DM2 with a disease duration of six months, using 2D echocardiograpy.

## Methods

Fifty-six patients (28 male, mean age 52.6 ± 6.5 years) with DM2, according to the American Diabetes Association (ADA) 2013 criteria, with a disease duration of a maximum of six months (recruited from the endocrinology and metabolism departments) and 56 age-matched healthy volunteers (24 male, mean age 50.1 ± 7.0 years) (recruited from the cardiology department) were included in the study.^15^ A detailed medical history, physical examination and 12-lead electrocardiography were obtained from the study population.

All subjects underwent a treadmill exercise test according to the Bruce protocol, or myocardial perfusion scintigrapyh to rule out latent ischaemia. Patients with evidence of ischaemia, arrhythmia on an electrocardiogram (ECG), LV dysfunction with an ejection fraction (EF) of < 50%, significant valvular disease, history of coronary artery disease, suspicion of secondary hypertension, uncontrolled hypertension, thyroid disorder, pulmonary disease and renal failure (defined as decreased glomerular filtration rate of < 60 ml/min/1.73 m^2^ for at least three months), type 1 DM, electrolyte imbalance, and technically insufficient echocardiographic and electrocardiographic data were excluded.

The local ethics committee approved the study. All participants provided written, informed consent prior to participation in the study.

Transthoracic echocardiographic examinations were performed using a commercially available cardiac ultrasound scanner (Acuson Sequoia 512 system with 2.5–4.0 MHz transducer, Siemens Mountain View, California, USA) in the left lateral position, according to the criteria of the American Society of Echocardiography.^16^ During echocardiography a continuous one-lead ECG recording was done.

Left ventricular end-diastolic and end-systolic volumes were determined in the apical view, and stroke volume and EF were measured using the modified Simpson’s equation.^16^ LV mass (LVM) was calculated with the Devereux formula as:

LVM (g) = 1.04 [(LVID + PWT + IVST)³ – LVID³] – 14

Where LVID = LV internal dimension; PWT = posterior wall thickness; IVST = interventricular septum thickness. LVM was indexed to body surface area (BSA) by dividing LVM by BSA.

Peak early diastolic (E) velocity, atrial contraction (A) velocity and E-wave deceleration time (DT) were measured from the transmitral pulsed-wave Doppler spectra, and the E/A ratio was calculated. Pulsed-wave tissue Doppler imaging (TDI) was performed in an apical four-chamber window with a sample volume of 5 mm and the monitor sweep speed was set at 100 mm/s to optimise the spectral display of myocardial velocities. All Doppler spectral velocities were averaged over three consecutive beats. The average pulsed-wave TDI-derived early (E′) diastolic myocardial velocity was obtained from the lateral and septal sides of the mitral annulus. Then the E/E′ ratio was calculated to provide an estimation of LV filling pressures.^17^ The TDI-derived late-diastolic wave (A′) was obtained from the mitral lateral annulus.

LA diameter was measured from the parasternal long axis with M-mode echocardiography. LA volumes were traced and calculated by means of the modified Simpson’s method from apical four- and two-chamber views, according to the guidelines of the American Society of Echocardiography and European Association of Cardiovascular Imaging.^16^ LA volumes were measured as: (1) just before the mitral valve opening, at end-systole (maximal LA volume or Vmax); (2) at the onset of the P wave on electrocardiography (pre-atrial contraction volume or Volp); and (3) at mitral valve closure, at end-diastole (minimal LA volume or Vmin). From these, the following measurements were calculated:

- LA passive emptying volume (PEV) = Vmax – Volp
- LA passive emptying fraction (PEF) = PEV/Vmax × 100
- LA active emptying volume (AEV) = Volp – Vmin
- LA active emptying fraction (AEF) = AEV/Volp × 100
- LA total emptying volume (TEV) = Vmax – Vmin
- LA total emptying fraction (TEF) = TEV/Vmax × 100

Left atrial volumes were indexed to BSA in all patients.^18^

## Statistical analysis

Statistical analyses were performed with the MedCalc Statistical Software version 12.7.7 (MedCal Software bvbv, Ostend, Belgium; 2013). All continuous variables are expressed as mean ± standard deviation and median (minimum–maximum). All categorical variables are defined as frequency and percentage. All continuous variables were checked with the Kolmogorov– Smirnov normality test to show their distributions. Continuous variables with normal distributions were compared using the unpaired Student’s t-test, while continuous variables with abnormal distributions were compared using the Mann–Whitney U-test. For categorical variables, the chi-squared test was used.

Pearson or Spearman’s correlation analyses were used to determine the associations between LA volume and function, and various laboratory parameters and 2D echocardiographic diastolic parameters. Multivariate evaluations were performed using linear regression analysis. The confounders that were found to have a statistically significant impact on the dependent variable on univariate analysis were described as the independent variables in a multivariate linear regression analysis model. The p-values less than 0.05 were considered significant.

Sample size justification: according to the article ‘Effects of diabetes mellitus on left atrial volume and functions in normotensive patients without symptomatic cardiovascular disease’,8 the Vmax value for DM2 patients was 40.9 ± 11.9 ml, and for the control group, 34.6 ± 9.3 ml. The mean difference was assumed as 6.3 ml; the standard deviation of the DM2 group was 11.9 ml and of the control group, 9.3 ml. With the assumption of 5% of type 1 error (a) and 80% power (1b), the sample size was calculated at 46 patients for each group. With a 20% drop-out rate, a minimum of 56 patients (112 in total) would have to be enrolled in the study.

## Results

The study population consisted of 112 subjects (52 male, mean age 51.7 ± 7.0 years). Patient characteristics, analysed according to the two groups, are shown in Table 1. The groups were similar regarding age and gender. In the DM2 group, 44 (78.6%) patients were hypertensive and 33 (58.9%) were receiving insulin and oral antidiabetic agents. Patients in the DM2 group were also taking more medications, such as acetylsalicylic acid, angiotensin converting enzyme inhibitors, beta-blockers and statins than the control group.

**Table 1:** Demographic characteristics and laboratory parameters of the groups

*Characteristics*	*Control group (n = 56)*	*DM2 group (n = 56)*	*p-value*
Age, year	50.1 ± 7.0	52.6 ± 6.5	0.06
Male, n (%)	24 (42.9)	28 (50)	0.55
BMI (kg/m^2^)	22.5 ± 2.0	28.0 ± 4.9	< 0.001
Tobacco use, n (%)	9 (16.1)	8 (14.3)	1.00
Hypertension, n (%)	6 (10.7)	44 (78.6)	< 0.001
Hyperlipidaemia, n (%)	11 (19.6)	47 (83.9)	< 0.001
Medication, n (%)			
ACE inhibitors	5 (8.9)	40 (71.4)	
Beta-blockers	1 (1.8)	16 (28.6)	
Statins	5 (8.9)	36 (64.3)	
ASA	37 (66.1)	3 (5.4)	
Insulin and OAD		33 (58.9)	
Fasting glucose (mg/dl)	93.9 ± 6.4	153.0 ± 67.0	< 0.001
(mmol/l)	(5.21 ± 0.36)	(8.49 ± 3.72)	
HbA_1c_ (%)	4.8 ± 0.6	8.1 ± 1.9	< 0.001
Total cholesterol (mg/dl)	211.4 ± 39.7	225.3 ± 50.6	0.11
(mmol/l)	(5.48 ± 1.03)	(5.84 ± 1.31)	
HDL-C (mg/dl)	48.2 ± 12.5	45.4 ± 8.5	0.16
mmol/l)	(1.25 ± 0.32)	(1.18 ± 0.22)	
LDL-C (mg/dl)	132.9 ± 38.2	140.1 ± 40.7	0.34
(mmol/l)	(3.44 ± 0.99)	(3.63 ± 1.05)	
TG (mg/dl)	141.0 ± 84.7	190.4 ± 105.0	0.01
(mmol/l)	(1.59 ± 0.96)	(2.15 ± 1.19)	
hsCRP (mg/l)	1.9 ± 1.2	5.3 ± 2.9	< 0.001
Uric acid (mg/dl)	4.6 ± 1.0	6.2 ± 1.6	< 0.001

DM: diabetes mellitus, BMI: body mass index, ACE: angiotensin converting enzyme, ASA: acetylsalisilic asid, OAD: oral antidiabetics, HbA_1c_: glycosylated haemoglobin, HDL-C: high-density lipoprotein cholesterol, LDL-C: low-density lipoprotein cholesterol, TG: triglycerides, hsCRP: high-sensitivity C-reactive protein.

Body mass index (BMI) and levels of triglycerides (TG), highsensitivity C-reactive protein (hsCRP), uric acid, fasting glucose and HbA_1c_ were significantly higher in the DM2 group compared with the control group (p < 0.05). There were no significant differences regarding total cholesterol and low- (LDL) and highdensity lipoprotein (HDL) cholesterol levels between the groups (p > 0.05) (Table 1).

Table 2 reports the results of 2D echocardiographic parameters reflecting diastolic function with preserved systolic function. Twelve (21.4%) subjects in the control group and 29 (51.8%) patients in the DM2 group had some degree of diastolic dysfunction. Mitral A wave, E/E′ ratio and mitral A′ wave were significantly higher, and mitral E′ wave was significantly lower in the DM2 group compared with the controls (p < 0.05).

**Table 2:** Echocardiographic parameters of the study groups

*Parameters*	*Control group (n = 56)*	*DM2 group (n = 56)*	*p-value*
EF (%)	61.9 ± 5.0	60.6 ± 4.4	0.14
Left ventricular mass (g/m^2^)	93.2 ± 8.4	102.3 ± 8.0	< 0.001
Mitral E (cm/s)	79.1 ± 14.1	81.2 ± 16.7	0.47
Mitral A (cm/s)	66.4 ± 13.2	80.8 ± 18.8	< 0.001
E/A ratio (cm/s)	1.2 ± 0.3	1.2 ± 0.9	0.68
Deceleration time (s)	199.0 ± 17.9	222.8 ± 19.7	< 0.001
Mitral E′ (cm/s)	18.5 ± 4.3	15.3 ± 3.3	< 0.001
Mitral A′ (cm/s)	14.0 ± 3.2	16.1 ± 5.0	0.011
E/E′ ratio (cm/s)	4.4 ± 1.0	5.5 ± 1.7	< 0.001
Diastolic dysfunction, n (%)	12 (21.4)	29 (51.8)	0.002

DM: diabetes mellitus; EF: ejection fraction.

There were no significant differences between the groups regarding EF, mitral E wave and E/A ratio (p > 0.05). LA diameter, and indexed Vmax, Volp, Vmin, AEV and TEV were found to be significantly higher in the DM2 group compared with the controls (p < 0.05). PEF was significantly lower in the DM2 group compared with the controls (p < 0.05). Between the two groups, there were no significant differences in indexed PEV, AEF and TEF (p > 0.05) (Table 3).

**Table 3:** The echocardiographic parameters for the LA function of the study groups

*Parameters*	*Control group (n = 56)*	*DM2 group (n = 56)*	*p-value*
LA diameter (mm)	33.3 (26–46)	37.5 (27–56)	< 0.001
Indexed Vmax (ml/m²)	19.8 ± 4.6	24.8 ± 6.6	< 0.001
Indexed Volp (ml/m²)	11.8 (4.6–23.6)	16.1 (9.5–30)	< 0.001
Indexed Vmin (ml/m²)	7.2 (2.8–14.0)	9.5 (3.8–24.5)	< 0.001
Indexed PEV (ml/m²)	7.4 ± 3.4	7.5 ± 3.2	0.66
Indexed AEV (ml/m²)	5.0 (0.7–16.4)	6.6 (2.4–15.1)	< 0.001
Indexed TEV (ml/m²)	12.5 ± 3.7	14.6 ± 4.1	0.004
LA passive emptying fraction (%)	35.5 ± 14.4	30.0 ± 11.1	0.003
LA active emptying fraction (%)	39.9 ± 13.5	42.0 ± 11.8	0.386
LA total emptying fraction (%)	60 (33.8–76.1)	63.9 (29.0–81.8)	0.05

DM: diabetes mellitus, LA: left atrium, PEV: passive emptying volume, AEV: active emptying volume, TEV: total emptying volume.

Patients in the DM2 group were divided according to presence of diastolic dysfunction. There were no significant differences within the DM2 group regarding LA volume and function (p > 0.05) (Table 4).

**Table 4:** Comparison of echocardiographic parameters regarding diastolic dysfunction for the LA function in the DM2 group

*Parameters*	*Diastolic dysfunction (+) (n = 29)*	*Diastolic dysfunction (–) (n = 27)*	*p-value*
LA diameter (mm)	37.4 ± 5.1	36.5 ± 5.8	0.548
Indexed Vmax (ml/m²)	25.8 ± 6.9	23.5 ± 6.2	0.196
Indexed Volp (ml/m²)	18.1 ± 5.8	16.1 ± 4.7	0.168
Indexed Vmin (ml/m²)	10.8 ± 4.6	9.2 ± 3.7	0.168
Indexed PEV (ml/m²)	7.6 ± 3.2	7.3 ± 3.4	0.735
Indexed AEV (ml/m²)	7.3 ± 2.8	6.8 ± 2.6	0.555
Indexed TEV (ml/m²)	14.9 ± 4.1	14.2 ± 4.0	0.505
LA passive emptying fraction (%)	29.5 ± 10.9	30.5 ± 11.5	0.751
LA active emptying fraction (%)	41.1 ± 11.1	43.0 ± 12.7	0.541
LA total emptying fraction (%)	58.7 ± 9.8	60.9 ± 9.4	0.402

DM: diabetes mellitus, LA: left atrium, PEV: passive emptying volume, AEV: active emptying volume, TEV: total emptying volume.

To determine the influential factors for LA volume, we examined the potential variables that we thought to be echocardiographically and clinically relevant: mitral A wave, E′ wave, A′ wave, E/E′ ratio, BMI, and fasting glucose, HbA_1c_, hsCRP and uric acid levels. There were weak positive correlations between all indexed LA volumetric parameters and all the variables except for indexed PEV and BMI, fasting glucose, HbA_1c_, hsCRP and uric acid levels, mitral A wave, E/E′ ratio and mitral A′ wave. There was a weak negative correlation between all indexed LA volumetric parameters and all the variables except indexed PEV and mitral E′ wave (Table 5).

**Table 5:** Correlation analysis of LA volume and function with 2D echocardiographic parameters and laboratory findings

		*Indexed Vmax (ml/m²)*	*Indexed Volp (ml/m²)*	*Indexed Vmin (ml/m²)*	*Indexed PEV (ml/m²)*	*Indexed >AEV (ml/m²)*	*Indexed TEV (ml/m²)*
Glucose (mg/dl)	r	0.153	0.252	0.182	-0.034	0.204	0.075
	p	0.108	0.007	0.055	0.725	0.031	0.429
HbA_1c_ (%)	r	0.288	0.367	0.294	0.006	0.301	0.192
	p	0.002	< 0.001	0.002	0.954	0.001	0.043
BMI (kg/m^2^)	r	0.430	0.441	0.368	0.135	0.340	0.325
	p	< 0.001	< 0.001	< 0.001	0.154	< 0.001	< 0.001
TG (mg/dl)	r	0.152	0.248	0.136	–0.047	0.239	0.089
	p	0.110	0.008	0.153	0.625	0.011	0.350
hsCRP (mg/l)	r	0.412	0.420	0.320	0.103	0.371	0.308
	p	< 0.001	< 0.001	0.001	0.281	< 0.001	0.001
Uric acid	r	0.362	0.378	0.297	0.125	0.283	0.253
(mg/dl)	p	< 0.001	< 0.001	0.001	0.190	0.002	0.007
Mitral A (cm/s)	r	0.328	0.380	0.292	–0.002	0.321	0.232
	p	< 0.001	< 0.001	0.002	0.981	0.001	0.014
Mitral E′ (cm/s)	r	–0.274	–0.258	–0.211	–0.094	–0.202	–0.226
	p	0.003	0.006	0.026	0.323	0.033	0.017
Mitral A′ (cm/s)	r	0.278	0.281	0.310	0.064	0.117	0.138
	p	0.003	0.003	0.001	0.504	0.220	0.147
E/E′ ratio (cm/s)	r	0.279	0.286	0.255	0.059	0.197	0.192
	p	0.003	0.002	0.007	0.539	0.037	0.028
E/A ratio (cm/s)	r	0.085	0.129	0.288	–0.050	–0.135	–0.140
	p	0.374	0.177	0.002	0.604	0.154	0.142

LA: left atrium, BMI: body mass index, TG: triglycerides, hsCRP: high-sensitivity C-reactive protein, PEV: passive emptying volume, AEV: active emptying volume, TEV: total emptying volume.

Univariate analysis showed that DM2, hypertension, age, BMI, and hsCRP and uric acid levels had a statistically significant impact on LA diameter, and indexed Vmax, Volp, Vmin, AEV and TEV. According to multivariate analysis when adjusted with other confounders, hypertension, age and BMI had a statistically significant effect on LA diameter; age and BMI had a statistically significant effect on indexed Vmax; age, BMI and uric acid level had a statistically significant effect on indexed Volp; uric acid level had a statistically significant effect on indexed Vmin; age had a statistically significant effect on indexed AEV; and age and BMI had a statistically significant effect on indexed TEV (Table 6).

**Table 6:** Univariate and multivariate analysis for predictors of LA volume and function of the study population

	*Univariate analysis*						*Multivariate analysis*							
*Parameters*	*DM2*	*HT*	*HL*	*Age*	*BMI*	*hsCRP*	*Uric acid*	*DM*	*HT*	*HL*	*Age*	*BMI*	*hsCRP*	*Uric acid*
LA diameter (mm)	< 0.001	< 0.001	0.028^1^	< 0.001	< 0.001	0.003	0.001	0.227	0.001	0.005	0.002	< 0.001	0.879	0.194
Indexed Vmax (ml/m²)	< 0.001	< 0.001	0.003	< 0.001	< 0.001	< 0.001	< 0.001	0.438	0.056	0.100	0.001	0.004	0.191	0.064
Indexed Volp (ml/m²)	< 0.001	< 0.001	< 0.001	< 0.001	< 0.001	< 0.001	< 0.001	0.991	0.181	0.244	0.003	0.016	0.226	0.042
Indexed Vmin (ml/m²)	< 0.001	< 0.001	0.007	< 0.001	< 0.001	0.001	0.001	0.869	0.171	0.334	0.069	0.099	0.371	0.034
Indexed PEV (ml/m²)	0.66	0.268	0.971	0.171	0.164	0.281	0.190	–	–	–	–	–	–	–
Indexed AEV (ml/m²)	< 0.001	< 0.001	0.001	0.001	< 0.001	< 0.001	0.002	0.822	0.623	0.476	0.010	0.064	0.383	0.486
Indexed TEV (ml/m²)	0.004	0.001	0.051	< 0.001	< 0.001	0.001	0.007	0.189	0.259	–	0.003	0.020	0.443	0.418
LA passive emptying fraction (%)	0.003	0.052	0.011	0.169	0.044	0.065	0.338	0.150	–	0.438	–	0.897	–	–
LA active emptying fraction (%)	0.386	0.769	0.499	0.393	0.718	0.430	–	–	–	–	–	–	–	–
LA total emptying fraction (%)	0.05	0.117	0.162	0.293	0.148	0.395	0.363	–	–	–	–	–	–	–

DM: diabetes mellitus, HT: hypertension, HL: hyperlipidaemia, BMI: body mass index, hsCRP: high-sensitivity C-reactive protein, LA: left atrium, PEV: passive emptying volume, AEV: active emptying volume, TEV: total emptying volume.

## Discussion

Diabetes mellitus can lead to changes in LA volume and function. In most studies, LA function is determined by performing realtime three-dimensional (3D) echocardiography, cardiac magnetic resonance imaging (CMRI), and strain and strain rate tests. However, in general practice, LA function can be easily and non-invasively determined by performing 2D echocardiography. In our study, we showed that even if LA size and volume were within normal limits, LA dysfunction may be present in patients with DM2 who was diagnosed in the preceding six months, and this finding was mainly due to BMI and age.

Recent studies have shown that LA enlargement, obtained from 2D echocardiography, is a good predictor of cardiovascular outcomes.^7^ However, there are several limitations to estimating LA size because of the irregular geometry of the left atrium. Additionally, the left atrium often enlarges asymmetrically, which causes underestimation of its size. Therefore, it has been suggested that LA volume may be a superior measure of LA size.^7^ Moreover, changes in LA volume are increasingly becoming a parameter of interest as a marker of overall cardiac function.

Several studies have shown that changes in LA size and mechanical function may be associated with adverse clinical events such as atrial fibrillation, stroke, diastolic dysfunction and LV failure, both in the general and the diabetic population.^6^,^8^,^10^-^14^,^19^,^20^ Moreover, it has been reported that indexed Vmax ≥ 32 ml/m^2^ predicts cardiovascular mortality and morbidity independently of myocardial perfusion sintigraphy-detected myocardial ischaemia with a six-year follow-up period.^21^

Cardiovascular imaging modalities for the determination of LA function, such as computed tomography (CT), CMRI, 2D and 3D echocardiography, are evolving. Although the main advantage of CMRI and CT over echocardiography is the determination of all parts of the left atrium, including the LA appendage, the use of iodine and radiation during CT and the usefulness of CMRI in patients with pacemakers limit their usage.^7^ Therefore, we preferred to use 2D echocardiography, which is a non-invasive, easy-to-use and accessible method to evaluate LA volume and function. Moreover, similar to our findings, the mean indexed Vmax value was 23.6 ± 5.8 ml/m^2^ in a newly diagnosed diabetes group in the study population of Zoppini.^14^

The incidence of diastolic dysfunction in patients with DM2 is reported to be 43 to 75%.^4^ Recent evidence suggests that LA dilatation and dysfunction may be a co-existing marker of diastolic dysfunction in patients with DM2.^4^ However, Kadappu et al. demonstrated LA dilatation may be present in patients with DM2 independent of diastolic dysfunction and associated hypertension.^4^ Recently, another study by Zoppini et al. reported that diabetes itself might cause LA enlargement.^14^ These findings suggest that co-existing diabetic atrial cardiomyopathy may independently alter the LA size and function.^4^,^14^

In our study, 51.8% of the diabetic patients had some degree of diastolic dysfunction with no difference regarding LA volume and function, compared with the diabetic patients without diastolic dysfunction. This finding and a weak correlation between 2D echocardiographic diastolic parameters and LA volume in our study may have been due to the duration of DM2, normal LV filling pressures determined by E/E′ ratio, and normal LV mass.

We demonstrated that increasing age and BMI had a significant effect on LA volume. The main difference of our study from previous ones was the duration of DM2, which was strongy and positively associated with larger LA diameter and impaired LA function. CARDIA investigators showed a 20-year follow-up period of diabetes was associated with indexed LA .diameters.^19^ On the other hand, Zoppini et al. showed a possible 65% LA enlargement (defined as indexed Vmax ≥ 34 ml/m^2^) for each 10 years’ duration of diabetes.^14^ On the basis of these findings, we speculate that although diabetes was an independent predictor of LA volume in univariate analysis, in multivariate analysis, age and BMI were the independent predictors of LA volume in the early stages of diabetes.

LA function is evaluated and indexed to BSA by calculating PEV, AEV, TEV and PEF, AEF and TEF from Vmax, Vmin and Volp. TEV describes the reservoir, PEV describes the conduit, and AEV describes the pump function of the left atrium. Contrary to current knowledge, Vmin increases, even in mild LV diastolic dysfunction, whereas Vmax increases in the later stages, suggesting that Vmin may be a more sensitive marker of LV diastolic dysfunction. Moreover, this finding underlines the importance of evaluation of LA function.^22^

Based on current knowledge, LA reservoir function is associated with worsening LV diastolic function.^7^ Graca et al. showed that LA reservoir and conduit function were reduced in asymptomatic DM2 patients.^23^ The same study also demonstrated that DM2 was independently associated with LA reservoir function, but not with conduit function.^23^

Mondillo et al. investigated only diabetic patients with normal LA size and did not find any difference in conduit and pump function. However, they showed LA deformation was impaired in diabetics even if LA volumes were similar between the groups.^24^ Murakana et al. showed decreased LA reservoir and conduit functions in patients with DM2 even in the absence of LA dilatation.^5^ Huang et al. demonstrated, with 2D echocardiographic evaluation, increased reservoir and pump function and reduced conduit function in patients with DM2.^6^ Recently, Atas et al. reported depressed reservoir and pump function with similar conduit function in patients with DM2 compared to the control group.^8^

In our study, in accordance with the study of Huang et al., we found reduced conduit, and increased pump and reservoir function in diabetic patients compared with the controls. The possibly inconsistent results with previous studies may have been due to different cardiovascular imaging techiques used for the determination of LA function, small sample sizes, different baseline characteristics, and different diabetes durations of the study populations.

There are some limitations to our study. As this was a crosssectional study, follow up of the patients for clinical endpoints such as AF and heart failure could not be done. Therefore, our study results cannot be used to direct standard clinical care. Moreover, as the population size was relatively small, our study does not permit any causal inferences and analysis on the effect of medications on LA volume and function. For this reason, long‑term follow up and large‑scale prospective studies are needed to determine the clinical predictive value of early LA functional impairment in this population. Evaluation of LA volume and function with 2D echocardiography was an additional limitation of our study.

## Conclusion

The results of our study showed impaired LA function may be present in patients with DM2 with a disease duration of a maximum of six months. BMI and increased age caus LA enlargement and LA volumes that were independent of the effects of hypertension and DM2. Further studies with larger sample sizes are needed to better define the underlying mechanisms.
